# Warm, not cold temperatures contributed to a Late Miocene reef decline in the Coral Sea

**DOI:** 10.1038/s41598-023-31034-8

**Published:** 2023-03-10

**Authors:** Benjamin Petrick, Lars Reuning, Gerald Auer, Yige Zhang, Miriam Pfeiffer, Lorenz Schwark

**Affiliations:** 1 Institute of Geosciences, Christian-Albrechts-Universität zu Kiel, Ludewig-Meyn-Straße 10, 24118 Kiel, Germany; 2 Institute of Earth Sciences, NAWI Graz Geocenter, University of Graz, Heinrichstrasse 26, 8010 Graz, Austria; 3 Department of Oceanography, Texas A&M University, College Station, TX 77843 USA

**Keywords:** Biogeochemistry, Marine biology, Palaeoceanography

## Abstract

Evidence shows that in the modern ocean, coral reefs are disappearing, and these losses are tied to climate change. However, research also shows that coral reefs can adapt rapidly to changing conditions leading some researchers to suggest that some reef systems will survive future climate change through adaptation. It is known that there were changes in the area covered by coral reefs in the past. Therefore, it is important to investigate the long-term response of coral reefs to environmental changes and high sea-surface temperatures (SSTs). However, because of diagenetic issues with SST proxies in neritic, metastable carbonate-rich environments, there is an incomplete and sometimes even incorrect understanding of how changes in SSTs affect carbonate reef systems. A good example is the Queensland Plateau offshore northeast Australia next to the threatened Great Barrier Reef. In the Late Miocene, between 11 and 7 Ma, a partial drowning caused the reef area on the Queensland Plateau to decline by ~ 50% leading to a Late Miocene change in platform geometry from a reef rimmed platform to a carbonate ramp. This reef decline was interpreted to be the result of SSTs at the lower limit of the modern reef growth window (20–18 °C). This article presents a new Late Miocene—ased SST record from the Coral Sea based on the TEX_86_^H^ molecular paleothermometer, challenging this long held view. Our new record indicates warm tropical SSTs (27–32 °C) at the upper end of the modern reef growth window. We suggest that the observed temperatures potentially exceeded the optimal calcification temperatures of corals. In combination with a low aragonite supersaturation in the ocean, this could have reduced coral growth rates and ultimately lowered the aggradation potential of the reef system. These sub-optimal growth rates could have made the coral reefs more susceptible to other stressors, such as relative sea-level rise and/or changes in currents leading to reef drowning. Given that these changes affected coral reefs that were likely adapted to high temperature/low aragonite saturation conditions suggests that reefs that have adapted to non-ideal conditions may still be susceptible to future climate changes due to the interaction of multiple stressors associated with climate change.

## Introduction

According to the Intergovernmental Panel on Climate Change, coral reefs are considered to be one of the most susceptible environments to future climate change^1^. Corals provide key ecosystem services and are the highest economic value environment due to a combination of benefits for tourism, fishing, and coastal protection, among other benefits^2, 3^. However, many of these reef systems are impacted by modern climate change^1^. This is true of the Australian Great Barrier Reef (GBR), which has shown repeated bleaching events over the last 10 years^4^. Yet at the same time, work from the Persian Gulf shows that corals can adapt to maximum monthly average SSTs around 34 °C in less than 6 ka^5–7^. Other studies have suggested coral reef adaptions to other aspects of future climate change, including acidification and changing sea levels^8–10^. This has led to a debate about the future of coral reefs between those who argue that adaptation will allow at least some reef systems to survive^8^ and those that argue that multiple stressors will lead to complete coral reef loss^11^. Resolving this debate is difficult as coral reef systems normally develop and decline on geological time scales beyond direct human observation. Therefore, it is important to understand what caused coral reef loss in the past, especially during periods where SSTs were higher than today. However, our understanding of how climate changes influenced reef systems in the past is poor.

A key challenge is that many quantitative geochemical proxies do not work on and in the vicinity of carbonate platforms, especially on multi-million-year time scales. This is because of issues with carbonate diagenesis, low total organic matter content, and a lack of long drill cores from reefs. Therefore, even though the timing of the loss of coral reefs and degradation of carbonate platforms is known during periods of the past, tying these changes to climatic change has been difficult^12^. This has led to a distorted view of the causes of coral loss in the past and our poor understanding of how changes in multiple environmental stressors affect coral reef systems and carbonate platforms over geological time scales.

The Queensland Plateau forms an isolated plateau offshore the GBR in the Coral Sea. Today it hosts several small pinnacle reefs and larger reef complexes, which together occupy 10–15% of its surface^13^. The modern reefs are relics of an Early to Middle Miocene (20–11 Ma) reef rimmed platform which covered ~ 64,000 km^2^ ^14^. In the Late Miocene, between 11 and 7 Ma, a partial drowning resulted in a decline of the reef area by 50%^15^. This change was accompanied by a shift in platform geometry from a reef-rimmed platform to a carbonate ramp during the Late Miocene^16^.

**Figure 1:**
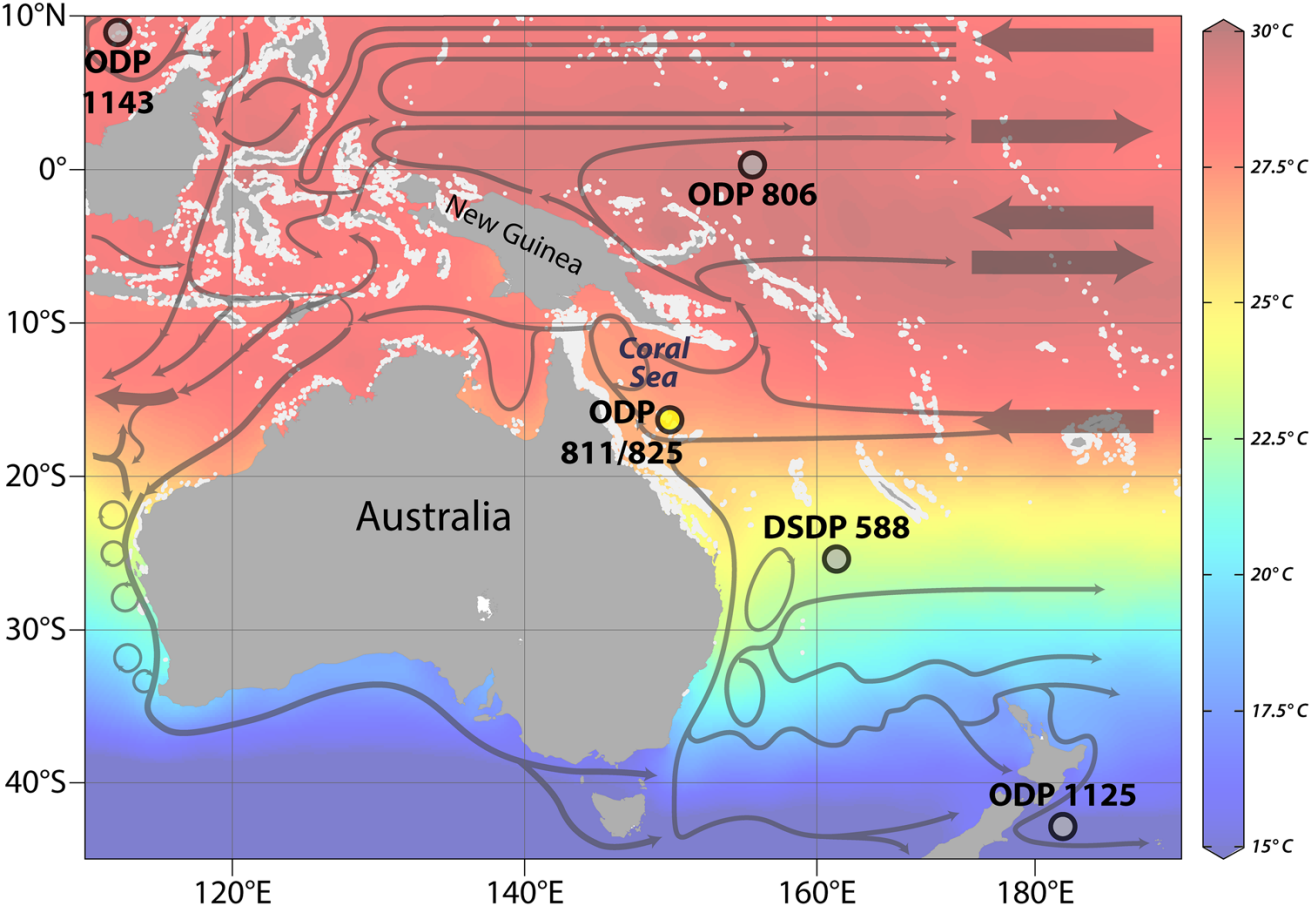
Location map of the Coral Sea and ODP Sites 811 and 825. Average sea surface temperatures (in °C) are based on the World Ocean Atlas 2018 (WOA18) annual mean temperature data, which is averaged from 1955 to 2010^21^. The SST color map was generated together with the base map in the program Ocean Data View^22^. The distribution of coral reefs, shown in white, is based on the dataset available from UN Environment Programme World Conservation Monitoring Centre^23^. Currents are simplified from^24^ and were adapted from^25^.

The Late Miocene has been suggested to be a time where average SSTs were similar to future climate change predicted for the end-of-century scenarios^17^. However, despite this, cool surface temperatures have been suggested as a reason for this dramatic reef decline, combined with other stressors such as changes in sea level and erosion^14, 15, 18, 19^. This interpretation is based on a δ^18^O_*Globigerinoides ruber*_ SST-reconstruction from ODP Site 811 on the Queensland Plateau (Fig. 1)^15^, which suggests SSTs ranged from 18 to 20 °C during the Late Miocene. These SST values are at the lower limit of the temperature range that supports reef growth^20^. Therefore, there is a disconnect between the high global SSTs and the temperatures reported from the Coral Sea during the Late Miocene. This study will re-evaluate the paleotempurature record from ODP site 811.

Studies using up-to-date methods have found temperatures at many other sites to be warmer than the original δ^18^O SST reconstructions indicated^26, 27^. Detailed studies found that δ^18^O SST estimates were about 10 °C too cold for Miocene tropical environments when using non-glassy foraminifera due to the alteration of their calcitic tests^28^. An alkenone-based U^K^_37_’ SST record from the upper continental slope just offshore the GBR in the Coral Sea, showed a very different trend to a δ^18^O glacial/interglacial record that was assumed to record SST^29^. However, even when knowing that the original δ^18^O records are problematic it remains a challenge to reconstruct SSTs in environments close to carbonate platforms that are rich in metastable carbonate phases with high diagenetic potential, such as aragonite. This is due to the influence of diagenetic phases in these environments, which can hamper the use of proxies such as δ^18^O, clumped isotopes, or Mg/Ca from carbonate archives^12, 29, 30^. The use of alkenone-based proxies such as U^K^_37_’ is limited to sea surface temperature < 29 °C and is poor at recording SSTs above 27 °C^31^, a temperature that can be exceeded in a tropical environment typical for reef growth. Therefore, a new proxy is needed to test the impact of SSTs on coral reefs in the past.

TEX_86-_based reconstructions can provide a better estimate of SST in these diagenetically active, metastable carbonate-rich environments. This is because TEX_86_ is based on the lipid composition of membranes from ﻿Thaumarchaeota found in many different parts of the ocean, and has been found including in low-productivity environments^32^. Because TEX_86_ is based on lipid biomarkers from a non-calcite organism, it is not susceptible to carbonate alteration or dissolution like other SST proxies^33^. Also, TEX_86_ is one of the few biomarker indexes that record temperatures above 30 °C, which is vital for reconstructing SSTs in tropical environments^34^. The biggest issue in the past has been the typically low organic matter content values in periplatform settings and questions about the accuracy of TEX_86_. However, large sample sizes, new, more sensitive methodologies, and a better understanding of the depth of production for TEX_86_ data have improved these issues (See supplement Sect. 1 for more info). Therefore, TEX_86_ provides a unique opportunity to reevaluate whether the extensive coral reef system of the Queensland Plateau was lost due to cooling between 11 and 7 Ma when studies suggest the platform drowned^15^.

**Figure 2:**
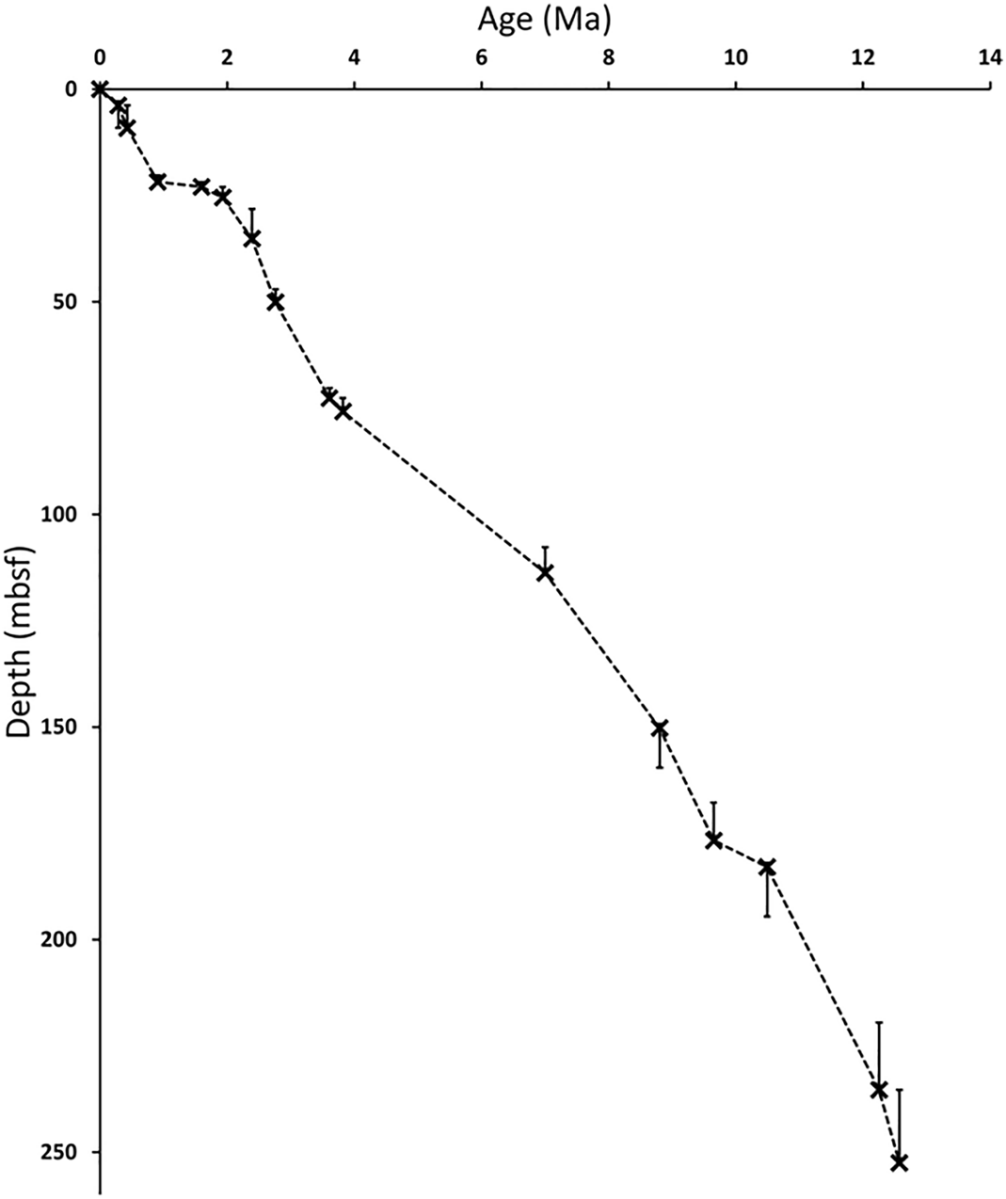
Age-depth model of ODP Site 811. Biostratigraphic markers are indicated as black crosses with potential depth errors shown as whiskers. Base occurrences are generally assumed to only have a positive depth (= deeper core depth) error while Top occurrences can only have a negative depth (= shallower core depths) error (see Tab. 1). Due to the nature of the last and first occurrences in the stratigraphic record.

ODP Site 811 (Fig. 1) was chosen for this project because it provides a continuous record of climate change from the Queensland Plateau for the last 11 Ma, as highlighted by our updated and improved age model (Fig. 2). We produced a new TEX86H-based biomarker SST record for ODP Site 811 from 11 to 7 Ma.

## Results and discussion

### Results

**Figure 3:**
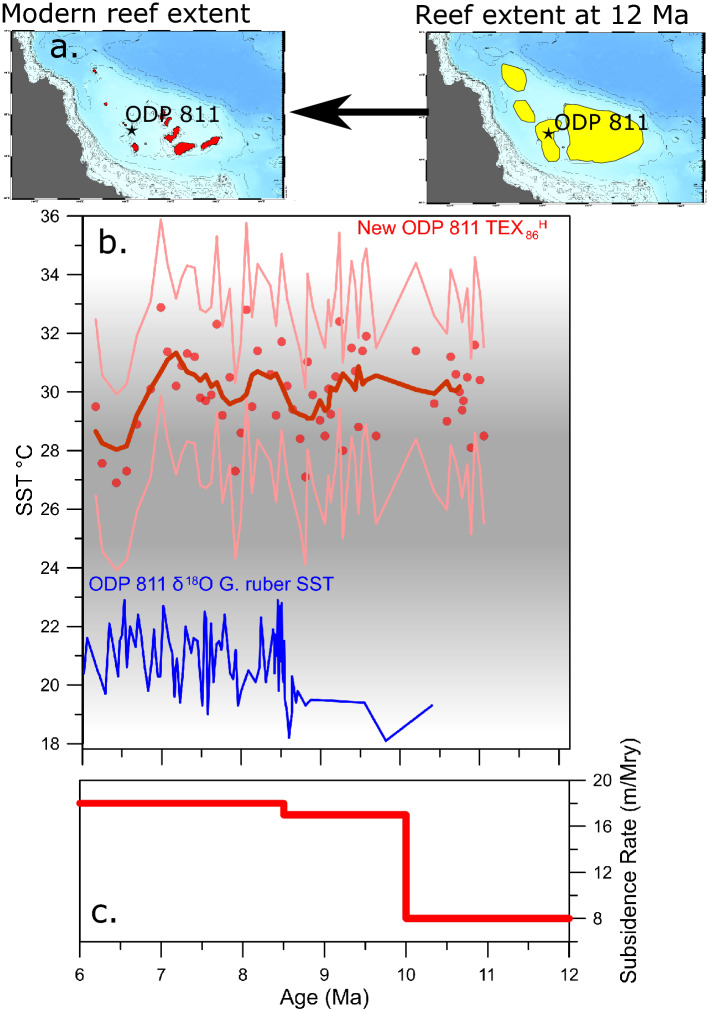
(**a**) maps of reefs before the late Miocene loss and the modern reef system on the Queensland Plateau based on reconstructions from^14, 15^ (**b**) New TEX_86_^H^ SST (red) and the previous *G. ruber* δ^18^O derived SST(blue)^15^. The raw data from the new TEX_86_^H^ record is shown by red dots, and a 7-point running average is shown by the thick red line. The 2-sigma error of the TEX_86_^H^ proxy is shown by the light pink lines. The temperature range of the modern “reef growth window” is shown in darker gray^41^. (**c**) Subsidence rates (red line) from^42^ for ODP Site 1193 on the Marion Plateau in the Coral Sea.

The TEX_86_^H^ derived SSTs at ODP Site 811, range from 32 to 27 °C, and were 9–12 °C warmer in the late Miocene than the δ^18^O-based SSTs previously published (Fig. 3). Based on running averages, there is no cooling or warming trend in the data overall (Fig. 3). The combined method and instrumental error of TEX_86_^H^ is 3 °C for each analysis, which, even at its maximum, does not encompass the old data. For data quality assurance, we ran several tests to determine if the TEX_86_ was influenced by non-thermal sources such as the Methane Index^35^and Ring Index^36^ or affected by outside sources using tests such as the BIT index^37^. Our work shows little evidence for a non-thermal input to the site. The BIT index is close to the quality threshold at the upper end of the section, but this only affects three data points. Overall, the tests show that the TEX_86_ at this site is being produced in optimal conditions for SST analysis and do not explain the difference between TEX_86_ and δ^18^O reconstructed temperatures. For a more detailed analysis of the TEX_86_ , please see supplemental Sect. 1. Average TEX_86_^H^-reconstructed SSTs were 29.7 °C, compared to the original δ^18^O-based SST average value of 20.8 °C^15^.

### Regional SST comparisons

**Figure 4:**
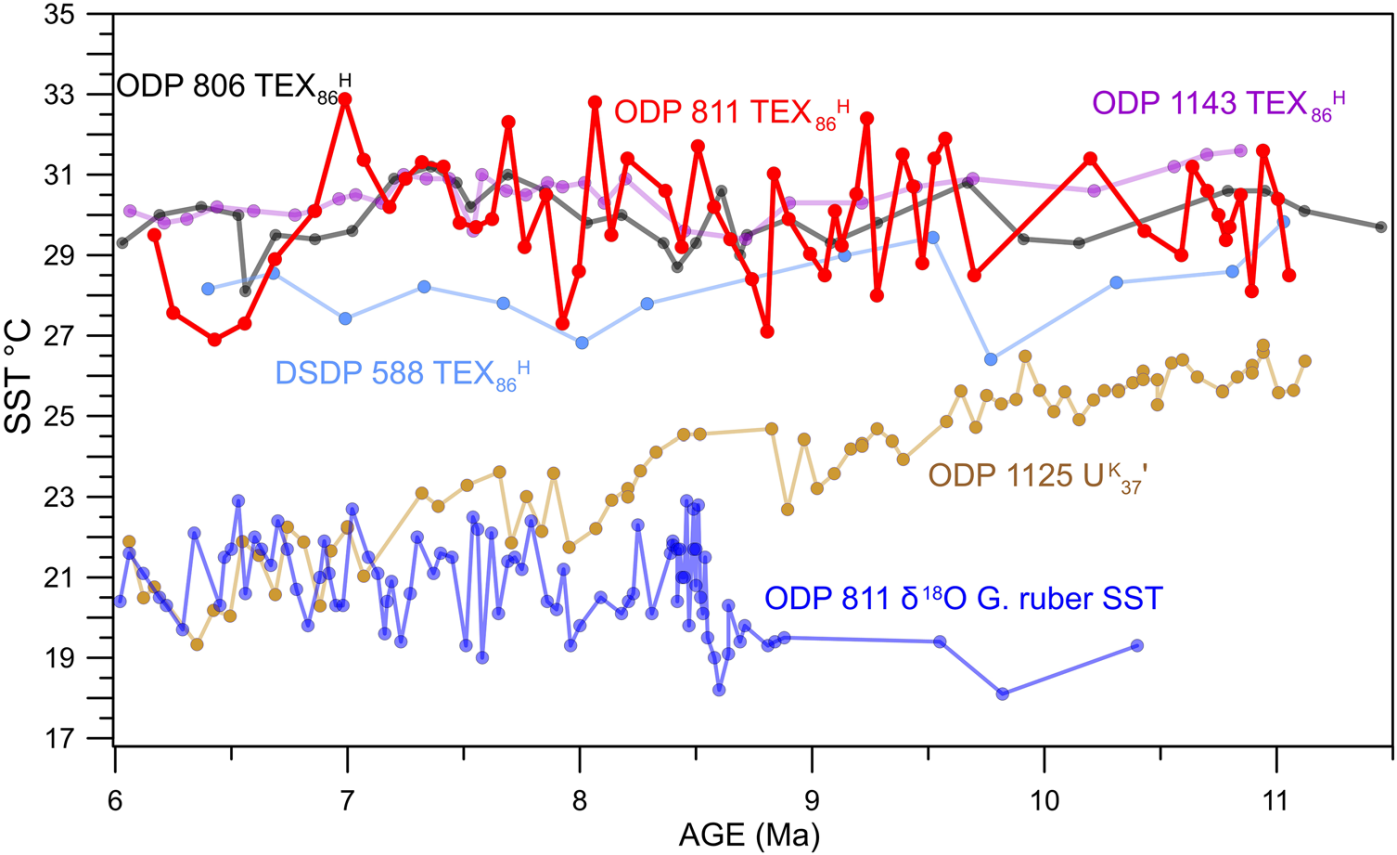
A comparison of different SST records from the western Pacific. The new 811 TEX_86_^H^ records from ODP site 811 (red) and the old δ^18^O record (dark blue)^15^ are compared to other Sites from the western Pacific (Fig. 1). These include ODP site 806 from the WPWP to the north of the Coral Sea (black), ODP site 1143 from the southern South China Sea (purple)^31^, and DSDP site 588 from the Lord Howe Rise at the southern limit of the Coral Sea (light blue)^38^ all of which are TEX_86_^H^ records. ODP site 1125 east of New Zealand south of the Tasman front (gold)^39^ is a U^K^_37_’ record. See Fig. 1 for locations of sites.

The TEX_86_^H^ SSTs are close to the average SSTs of 29.9 °C from ODP 806 in the modern West Pacific Warm Pool over the same time interval (Figs. 1, 4). Comparing our record to other SST records from sites in the western Pacific warm pool, based on TEX_86_^H^ (Fig. 4) shows that the temperatures and trends are very similar to those seen in the West Pacific Warm Pool during the Late Miocene^31^ (Fig. 4).

They are slightly warmer than TEX_86_^H^ temperatures from DSDP site 588 south of the site at the southernmost limit of the Coral Sea at the Lord Howe Rise. Together with being slightly cooler than the sites further north suggests that the SSTs from ODP site 811 fall within the expected SST gradient^38^. However, the ODP site 811 SSTs we found are very different from the SSTs and cooling trends that were seen in most of the subtropical southern hemisphere during the Late Miocene^39^ (Fig. 4). The original δ^18^O SSTs are lower than the U^K^_37_’ temperatures at ODP site 1125 located just off of New Zeeland on Tasman Front during this time further suggesting that these SSTs do not fit with other SST reconstructions for this time^39^ (Fig. 4). This suggests that the Coral Sea was tropical rather than subtropical, as previously suggested^15^, during the Late Miocene. Given that the average SST at the Coral Sea today is between 24 and 25 °C^21^ (Fig. 1), Late Miocene temperatures were between 2 and 6 °C warmer than in the modern Coral Sea. During the Late Miocene, the site was only 3 degrees further south^40^, meaning that latitudinal drift would not explain the changes.

The TEX_86_^H^ SSTs are 9–12 °C warmer than the old δ^18^OSST record. Research showed that when “frosty” foraminifera showing authigenic calcite overgrowth, as opposed to glassy foraminifera, are used, the reconstructed δ^18^O temperatures are about 10 °C too cold^27^. This recrystallization is produced in the deep ocean and adds a benthic temperature component on top of the SST signature preserved in the test, causing the cold bias. The initial reports describe overgrowths and infillings as minimal^18^. While this would have been seen as well preserved at the time of publication, today, these foraminifera would be described as frosty, which is defined as ﻿foraminifera that has undergone relatively modest diagenetic alteration^27^. The presence of overgrowth has been confirmed by re-examining the foraminifera as part of this study. Therefore, despite the best attempts to avoid overgrowth effects, they are affecting the δ^18^O record. However, the inferred Late Miocene cooling and its effect on the reefs of the Queensland Plateau were not just based on the δ^18^O-SSTs but were supported by carbonate facies analysis.

### Re-analysis of carbonate facies

The reduction in coral reef platform area in the late Miocene was accompanied by a transition in carbonate platform morphology and biota on the analyzed southwestern margin of the Queensland Plateau^16^. The Middle Miocene rimmed carbonate platform was dominated by a tropical photozoan skeletal assemblage, including green algae, corals, and large benthic foraminifera. Bryozoans occur as a minor component. During the Late Miocene, the platform evolved into a carbonate ramp with a skeletal assemblage dominated by bryozoans and foraminifera, including abundant large benthic foraminifera but also planktonic foraminifera^16^. In contrast, corals and green algae are missing in these upper Miocene deposits. It was thought that the Late Miocene facies is more representative of a foramol association (sensu^43^)^16^. This shift in the skeletal assemblage was interpreted to reflect a transition from tropical to non-tropical, warm-temperate climate conditions^14–16, 18^, partly supported by the oxygen isotope record^15, 18^.

Additionally, a number of researchers^15^ have discussed the possibility that changes in nutrients influenced the facies transition on the Queensland Plateau. Based on paleontological data it was thought that an increased nutrient availability would have contributed to the Late Miocene facies transition^15^. Others in contrast argued that an influence of nutrients would be unlikely, partly based on the fact that large benthic foraminifera assemblages in mesotrophic environments typically would be rich in large imperforate forms while the Late Miocene carbonates from the Queensland Plateau are characterized by perforated forms^16^. However, it has been shown that perforate large benthic foraminifera are common in Miocene equatorial carbonates with foramol facies^44^. These carbonates were deposited under tropical temperatures where nutrient levels were in the high oligotrophy to mesotrophy range^44^. There is now a growing number of studies demonstrating that carbonate platforms dominated by foramol and other heterozoan skeletal grain associations were thriving under tropical temperature conditions during the Miocene^45–47^. All of these studies emphasized that carbonate grain associations on their own are insufficient to allow an unequivocal interpretation of paleoclimatic conditions. It has been stressed that other data, e.g. from climate proxy studies, are required to support an environmental interpretation^48^. Our presented new temperature proxy data presented here shows that environmental factors other than temperature are responsible for the observed carbonate facies change on the Queensland Plateau during the Miocene. A more detailed assessment of the paleontological and facies evidence for sea surface temperature conditions is given in the supplemental "Results and discussion" section.

### Implications for coral reef development

Our new TEX_86_^H^ SST data suggests a new driver is needed to explain the loss of corals. We find it is unlikely that cooler SSTs were responsible for the loss of these reefs, as suggested in the classical study of Isern et al.^15^. Our TEX_86_^H^ SST data fall within the upper end of the modern “reef growth window” during the time of the coral reef drowning, which defines the environmental conditions that are optimal for the development of reefs^41^. SSTs between 25° and 29 °C generally are considered to be ideal for reef development^41^, while winter temperatures of 18 °C define the lower limit^20^ (Fig. 3). An upper-temperature limit for modern reef growth is not well defined, but coral reefs in the Persian Gulf experience annual average temperatures of ~ 28 °C SST, but with summer temperatures exceeding 34 °C^5, 20^. Generally, the calcification and upward growth rates of many coral species increase linearly with annual SST^49, 50^. However, this trend seems to reverse above an annual temperature of 26–27 °C, with a reduction of the calcification and extension rate at higher temperatures^51^. There is pronounced taxonomic variation in the effect of temperature on corals, but a recent meta-analysis indicates that severe future temperature increases are expected to reduce their calcification rates and therefore could limit their capacity to build reefs^52^. Hence the observed Late Miocene average temperatures of ~ 30 °C (Fig. 3) might have induced stressed conditions in the coral reefs of the Queensland Plateau during the Late Miocene similar to what is occurring in the GBR in the modern-day.

Additionally, there might have been limited coral calcification and extension rates due to the relatively low aragonite supersaturation in the Mid-Late Miocene tropical ocean. Reconstructions indicate that the aragonite supersaturation in the tropical ocean was lower in the Late Miocene compared to pre-industrial values, with a minimum at ~ 9Ma^53^. This is similar to the modern ocean where acidification is predicted to impact future coral reef net carbonate production. It was observed that the calcification rate of reef corals was lower than present-day values for much of the Neogene, not only in marginal basins but also in examples from clear-water reef window-type environments^54^. This change is explained with the chronically low carbonate saturation state of the global ocean during this time. Therefore, much like in the modern ocean, the Late Miocene corals were experiencing high thermal conditions and low aragonite supersaturation. Environmental conditions such as temperature and aragonite supersaturation impact the rates of coral reef carbonate production and their capacity to accrete to sea level in many ways^55^. Bioerosion and synsedimentary carbonate dissolution are other important factors besides coral calcification rates. The response of coral reef net carbonate production to changes in temperature and aragonite saturation state is complex^55^. However, lower aragonite saturation states are not only expected to reduce coral calcification rates^52^ but also to increase bioerosion^56^ and reef carbonate dissolution^57^.

Yet despite these conditions, this did not lead to coral reef loss in the Coral Sea, and there is some evidence that the corals had adapted to the suboptimal conditions by 11 Ma. During the Mid-Miocene Climatic Optimum (MMCO), tropical ocean temperatures likely were similar to, or higher then SSTs during the late Miocene^58–60^, and the surface ocean aragonite saturation state was even lower compared to the Late Miocene^54^. Despite this, reef growth was abundant and even showed signs of expansion on the Queensland Plateau during the MMCO^16^. The corals that existed during the Late Miocene, as mentioned above, may have adapted to these MMCO conditions. This is similar to the modern where coral reefs can adapt to grow in suboptimal environments such as the Persian Gulf^5–7^. However, despite these adaptions, the coral reefs still declined between 11 and 7 Ma. Therefore, while warm SSTs and low aragonite saturation might have reduced net reef carbonate production, we believe that this alone could not have caused the dramatic loss of coral reefs.

Therefore, or study suggests that additional changes must have triggered the collapse of an adapted coral reef system. There are two main candidates for this: increased subsidence or changes in ocean circulation. Increased subsidence could have contributed to the drowning of the coral reefs in the Coral Sea^42^. Research showed an increase in subsidence on the Marion Plateau just south of the Queensland Plateau between 11 and 10 Ma, concurrent with the loss of the coral reefs. This was matched by high late Neogene subsidence rates seen on the NW shelf of Australia^61, 62^. Therefore, there is some evidence for increased subsidence rates over large parts of the northern Australian plate around 11 Ma. However, the subsidence rates were insufficient to outpace the aggradation potential of modern coral reefs^42^. Therefore, the combined effects of high subsidence rates, the lower aragonite supersaturation during the Late Miocene, and the higher SSTs we found in this study could have put the coral reefs at risk. This would have meant that the coral reefs were not able to keep up with the change to higher subsidence rates at around 10 Ma. Therefore, this event could have caused the coral reefs, which had adapted to high SSTs by reducing extension rates to drown.

An additional stressor could have been the intensification of open ocean currents during between 12 and 8 Ma^63^, which could have aggravated the Late Miocene coral reef demise in the Coral Sea^19^. There is evidence of shifting wind and current patterns during the late Miocene^63, 64^. It was hypothesized that cooler late Miocene SSTs at the Queensland Plateau could indicate an increased influence of southern sourced waters and intensification of the sub-thermocline water mixing^15^. This does not fit our SST record, and we do not see any evidence for long-term permanent SST cooling during 11 to 7 Ma when the coral reefs drowned (Figs. 2, 3) as would be expected with a change in currents. Even the coldest temperatures reconstructed at Site 811 are still in the tropical “coral reef window” (Figs. 2, 3). This observation is consistent with the limited data from the southern limit of the Coral Sea at ODP 588^38^.

It was suggested that a new wind-driven current system was established in the Coral Sea during the Late Miocene^19^. They argue that this could have caused topographical mixing and upwelling of nutrient-rich sub-surface waters on the leeward side of the Queensland Plateau, ultimately causing the backstepping of the leeward margin and the reduction in platform size. Additionally, they emphasize that strong current systems can have detrimental effects on carbonate platforms, e.g. through intensified erosion limiting their potential to aggrade and keep up with sea-level rise^19^. Therefore, whichever combination of stressors, it seems that synergetic stressors might explain the reduction in reef area in the Coral Sea during the Late Miocene.

In summary, our work shows that the long-held hypothesis that the Late Miocene carbonate platform drowning in the Coral Sea was caused by cooling must be revised. SSTs remained warm and even exceeded the optimal temperature range of modern coral reef growth during the entire period. Instead, we suggest that SSTs on the higher end of the modern reef growth window and the relatively low aragonite saturation state of the surface ocean made the coral reef more susceptible to other environmental stressors, such as the relative sea-level rise and increased erosion, and/or nutrient inputs from open ocean currents. The combination of stressors likely led to the partial drowning of the Queensland Plateau between 11 and 7 Ma.

Interestingly, these drivers are similar to those predicted for future climate change. Models show that SSTs and sea levels will increase with future climate change^65^. Furthermore, high CO_2_ levels are leading to increased acidification of the oceans, inhibiting carbonate production^65^. It may be that the coral reefs on the Queensland Plateau had successfully adapted to the climate changes experienced during the Mid-Miocene. However, these adaptations might have left the corals susceptible to changes in sea level and possibly changing nutrient conditions. Model studies show that modern coral reefs could survive various individual climate change effects either through adaptation or by migration to more protected environments^55^. However, combining the various stressors into a single model significantly reduces the survivability of coral reefs^55^. Our record shows that in combination with other factors, high SSTs can cause a significant decline of coral reefs with a similar set of conditions as predicted by models issued by the International Panel on Climate Change^55^. Therefore, our record shows that the loss of coral reefs in the Coral Sea during the Late Miocene was not a result of rapid cooling in SSTs but instead a combination of negative stresses that together lead to the observed dramatic reduction in reef area between 11 and 7 Ma.

## Methods

### Age model

The biostratigraphic age model of ODP Site 811/825 was reevaluated based on a semiquantitative study of 53 samples from the entire core including sections outside of the SST reconstructions done above (see supplementary data for details). Slides were prepared according to the drop technique (cf.^66^; see^67^ for details on the methodology). Samples were screened for nannofossils and their presence was recorded based on a distinction between few (F: one to five specimens per slide), rare (R: one specimen every other field view), common (C: one to five specimens per field view), abundant (A: more than 10 specimens per field view), and dominant (D: taxon occurs ubiquitously with only rare occurrence of other taxa present). The maximum potential depth error of the recorded nannofossil datums results from sample spacing between the unequivocal record of a taxon and its absence in the next sample above (for top occurrences: T), or below (for base occurrences: B). In the age-depth plot (Fig. 2), this uncertainty is also expressed as vertical error bars^68, 69^. The defined biostratigraphic events are summarized in Table 1 and the age-depth model is visualized in Fig. 4.

**Table 1:** Nannofossil biostratigraphic events detected during the semiquantitative screening of 53 samples (see supplementary material nannofossil data and depth uncertainty of the datums).

Nannofossil event	Age (Ma)	Depth (mbsf)	Depth error (mbsf)	Site	Reference
B. *Emiliania huxleyi*	0.29	3.82	5.32	ODP 811	Gradstein et al.^70^
T *Pseudoemiliania lacunosa*	0.43	9.13	5.32	ODP 811	Gradstein et al.^70^
T *Reticulofenestra asanoi*	0.91	21.82	1.52	ODP 811	Gradstein et al.^70^
T *Calcidiscus macintyrei*	1.60	22.99	1.18	ODP 811	Gradstein et al.^70^
T *Discoaster brouweri*	1.93	25.49	2.5	ODP 811	Gradstein et al.^70^
T *Discoaster pentaradiatus*	2.39	35.18	7.04	ODP 811	Gradstein et al.^70^
T *Discoaster tamalis*	2.76	50.10	3.02	ODP 811	Gradstein et al.^70^
T *Sphenolithus spp.*	3.61	72.63	2.38	ODP 811	Gradstein et al.^70^
T *Reticulofenestra pseudoumbilicus*	3.82	75.79	3.16	ODP 811	Gradstein et al.^70^
Ta *Reticulofenestra pseudoumiblicus*	7.10	113.79	6.12	ODP 811	Gradstein et al.^70^
Ba *Reticulofenestra pseudoumbilicus*	8.80	150.26	9.37	ODP 811	Gradstein et al.^70^
T *Discoaster hamatus*	9.61	176.7	8.9	ODP 811	Gradstein et al.^70^
B *Discoaster hamatus*	10.57	182.9	11.66	ODP 811	Gradstein et al.^70^
T *Coronocyclus nitescens*	12.25	235.35	15.82	ODP 825	Gradstein et al.^70^
Tc *Calcidiscus premacintyrei*	12.57	252.48	17.13	ODP 825	Gradstein et al.^70^

Ages are based on the most recent compilation in the Geologic Time Scale 2020^70^. *B* Base occurrence, *T* Top occurrence; *Ba* Base absence; *Ta* Top absence; *Tc* Top common cf^71, 72^.

### GDGTs

For this project, we took 50 cc of sediment leading to between 50 and 60 g of sediment being extracted. Pilot work showed that this was necessary to have enough material to measure SSTs on. Samples were Soxhlet extracted for 48 h using a solvent mixture of DCM: MeOH (9:1, v/v). Elemental sulfur was removed by the addition of activated copper turnings. Excess solvent was evaporated by a Büchi solvent evaporator to a final volume of 2 ml and samples were then transferred into a 4 ml vial, where the total extract (TE) was taken to dryness under a gentle stream of nitrogen. TEs were fractionated into aliphatic, aromatic, and polar fractions by silica gel-column chromatography (6 ml SPE column, 2.8 g Silica 60 mesh, 25–40 μm) using solvents with increasing polarity in an LC-TECH automated SPE system. NSO (polar) compounds were eluted with 14 ml DCM/ MeOH (1:1, v/v). The polar fraction was reconstituted in hexane/isopropanol (9:1, v/v) and re-chromatographed over aminopropyl-substituted silica gel (3 ml SPE column, 1.0 g aminopropyl-silica, 25–40 μm). The alcohol fraction containing the GDGTs was eluted with 5 ml of hexane/isopropanol (9:1, v/v) and after drying was re-dissolved in hexane/isopropanol (99:1, v/v) to a final concentration of 6 mg/ml for injection into the HPLC/MS system.

GDGTs were measured on an Alliance 2695 HPLC system (Waters, UK) coupled to a Micromass ZQ single quadrupole mass spectrometer following the analytical protocol of^73^. The HPLC instrument was equipped with two Waters BEH HILIC silica columns (2.1 × 150 mm; 1.7 µm particle size) and a guard column maintained at 30 °C. Target compounds were eluted with a flow rate of 0.2 ml/min starting isocratically with 82% eluent A (n-hexane) and 18% eluent B (n-hexane:2-propanol (9:1, v/v)) for 25 min. Thereafter, a linear gradient was set to 65% eluent A and 35% eluent B in 25 min, followed by a linear gradient to 0% eluent A and 100% eluent B in 30 min. Re-equilibration of the column afforded 82% eluent A and 18% eluent B for 20 min. Detection of isoprenoid GDGTs was achieved using a Micromass ZQ single quadrupole mass spectrometer (MS) equipped with an atmospheric pressure chemical ionization (APCI) interface operated in positive ion mode. MS settings were: source temperature, 150 °C; vaporizer temperature, 500 °C; corona, 2 µA; cone voltage, 40 V; extractor voltage, 3 V; radio frequency (RF) lens, 0.1 V; desolvation gas, N_2_ at 8 L/min. Detection of archaeal core lipids was achieved by single ion recording of their protonated molecular ions [M + H]^+^ (dwell time, 234 ms) and compounds were quantified by integration of peak areas using MassLynx© software. Calculation of TEX_86_ followed^74^ and TEX_86_^H^ followed^34^. Reproducibility upon duplicate measurements showed a relative standard error of < 2% and samples analyzed using both methods showed a relative standard error of < 3%.

## Supplementary Information

Supplementary Information 1.

Supplementary Information 2.

## Data Availability

All data needed to evaluate the conclusions in the paper are present in the paper and/or the Supplementary Materials. The datasets used and/or analyzed during the current study will be available from the corresponding author upon reasonable request. Requests for the data should be submitted to: benjamin.petrick@ifg.uni-kiel.de.
