# Supplementary material for: Warm, not cold temperatures contributed to a Late Miocene reef decline in the Coral Sea
Source: Sci Rep. 2023 Mar 10;13:4015. doi: 10.1038/s41598-023-31034-8 (PMC10006184; doi:10.1038/s41598-023-31034-8)
Supplement: Supplementary file 1 — Supplementary Information 1. [file 41598_2023_31034_MOESM1_ESM.docx]

**Supplementary Materials**

**1 TEX_86_ proxy justification**.

While there is some debate about the accuracy of TEX_86_ records in some situations, we are confident that our SST record is accurate for the Coral Sea. Initially, we note that the raw data GDGT passed all of the tests that have been proposed for detecting non-thermal inputs into the record ^1,2^. Therefore, we do not think that non-thermal inputs can explain the difference between the data produced in this study and previous records for the same site.

In this study, we report SSTs as TEX_86_^H^ for several reasons. First, most of the published records for this area are reported in TEX_86_^H^ facilitating a one-to-one comparison of the different records. Second, comparing TEX_86_^H^ to the other commonly used proxy calculation $\mathrm{TEX}_{86}^{\mathrm{BAYSPAR}}$ results in SSTs of almost the same values. The $\mathrm{TEX}_{86}^{\mathrm{BAYSPAR}}$ values are on average 1 degree warmer than the TEX_86_^H^ values. Therefore, this difference would not change the conclusions of our research and instead reinforce them. The only major difference is that the BAYSPAR reconstruction has error bars of 5 °C. This is because there are no records from the area to construct a local proxy calculation, and therefore the error bars are automatically set to maximum. We feel that, given that the two data sets show approximately the same temperature and just differ in the error, $\mathrm{TEX}_{86}^{H}$ provides the more scientifically robust error bars at the moment. We will provide the raw data to online databases, however, and, as more work is done in the study region, a more accurate calculation of the error may be possible. We also discuss possible sources of error below.

We removed several samples because of issues with the data. In three samples, we were not able to measure any GDGTs. In addition, three more samples were missing one of the isoprenoid GDGT compounds. This led to errors in several of the indices shown below and produced non-thermal SST values, so we removed these samples as well. We do not include these measurements in the discussion below because they were not able to provide accurate SST data.

To test the origins of the rest of the $\mathrm{TEX}_{86}^{H}$signal and whether there was any potential interference in the record, we performed several tests. The Methane Index (MI) is used to exclude any data affected by gas-hydrate-related anaerobic oxidation of methane ^3^. Our MI values are below the 0.5 value for rejection. We also used the GDGT0% index to eliminate any samples with a high amount of GDGTs originating from sedimentary archaeal methanogenesis ^4,5^. Once again, the values were well below the 67% cut-off for excessive methanogenesis. To test whether the samples had odd arrangements of compounds like those found in the Red Sea, we used the GDGT_RS_ % ^6^. Once again, the values were below the rejection value of 24%. We also used the ring index (RI) to evaluate whether the GDGTs deviate from modern values ^7^. All of our data fell within the acceptable error envelope of 0.3. Finally, we used the 2/3 index to make sure that the GDGTS were being formed on the surface ^8,9^. While the appropriate cutoff for this test is still being debated, our data values are low and seem to indicate surface production.

The one index that did raise issues was the BIT index. This index was developed to track the amount of terrestrial material that could interfere with the TEX_86_ values via soil-sourced GDGTs ^10^. The original cutoff point is 0.4, although there is a debate about whether that is a hard and fast cutoff ^10^. The other issue is that changes in Crenarcheol have been shown to affect the BIT index ^11^. Our data show a trend towards lower BIT values towards the top of the succession. However, all of the data, except for 3 data points, is below the 0.4 cutoff. Also, two of these points were close to the 0.4 cutoff. We have decided to keep all the points in our results because of the uncertainty of the meaning of the BIT index. However, some caution about the absolute values towards the top of the record should be noted.

We have also considered two other issues that could have affected the TEX_86_ data. The first issue is the depth of production. It is well known that TEX_86_ based SSTs can be affected by the fact that GDGTs are sometimes produced deeper in the water column, rather than at the surface. In other instances, the TEX_86_ represents an average of the top 100 m of the water column. Finally, there is some evidence that the depth of production can change even within one record ^12,13^. However, if, for any of these reasons, the temperatures we found were produced deeper in the water, this suggests that the surface temperatures were even warmer. Consequently, the potential deep-dwelling of archaea will not change our conclusion that the Coral Sea experienced warm tropical WPWP type temperatures during the Late Miocene. Furthermore, our SSTs are about the same or even slightly lower than others found in the area from the same time, such as at ODP 806 from the WPWP and ODP 1143 from the South China Sea ^14^. Finally, WOA data for the area around ODP 811 shows the change in temperature of the upper 100m is within 1 degree, meaning that it is likely that deeper produced GDGTs would still accurately reflect SSTs. All of this suggests that depth of production is not an issue with this TEX_86_ record.

A second issue with the data could be the effects of transportation. It is known that biomarkers can be transported laterally by currents ^15^. Furthermore, in these low TOC environments, there is a possibility that transported GDGTs are a larger contributor to the sediment than locally produced GDGTs. The connections between the Coral Sea and the WPWP at the present are minor and only affect the site during the local summer monsoon ^16,17^. This is why today ODP 806 has average SSTs of 29 °C, whereas at ODP 811 they are 25 °C ^18^. Therefore, if lateral transport of GDGTs will occur at ODP 811 today, most of the water would be coming directly from the South Equatorial Current west of the Coral Sea, which is an area with similar or colder SSTs ^16,17^. Most importantly, however, even if our SST record reflects warm water that was transported from warmer areas to the north, that still means that there was a stronger connection between the WPWP and the study site during the Late Miocene than there is today and that there was a strong tropical influence on the site throughout the Late Miocene.

Finally, while outside forces might have affected the TEX_86_^H^ at site ODP 811, none of them would be sufficiently significant to explain the large difference in temperatures between the TEX_86_ and δ^18^O data or to change our interpretation that the site was tropical throughout the Late Miocene. Therefore, we conclude that the TEX_86_ values found here suggest that a stable tropical environment existed between 11-6 Ma in the Coral Sea.


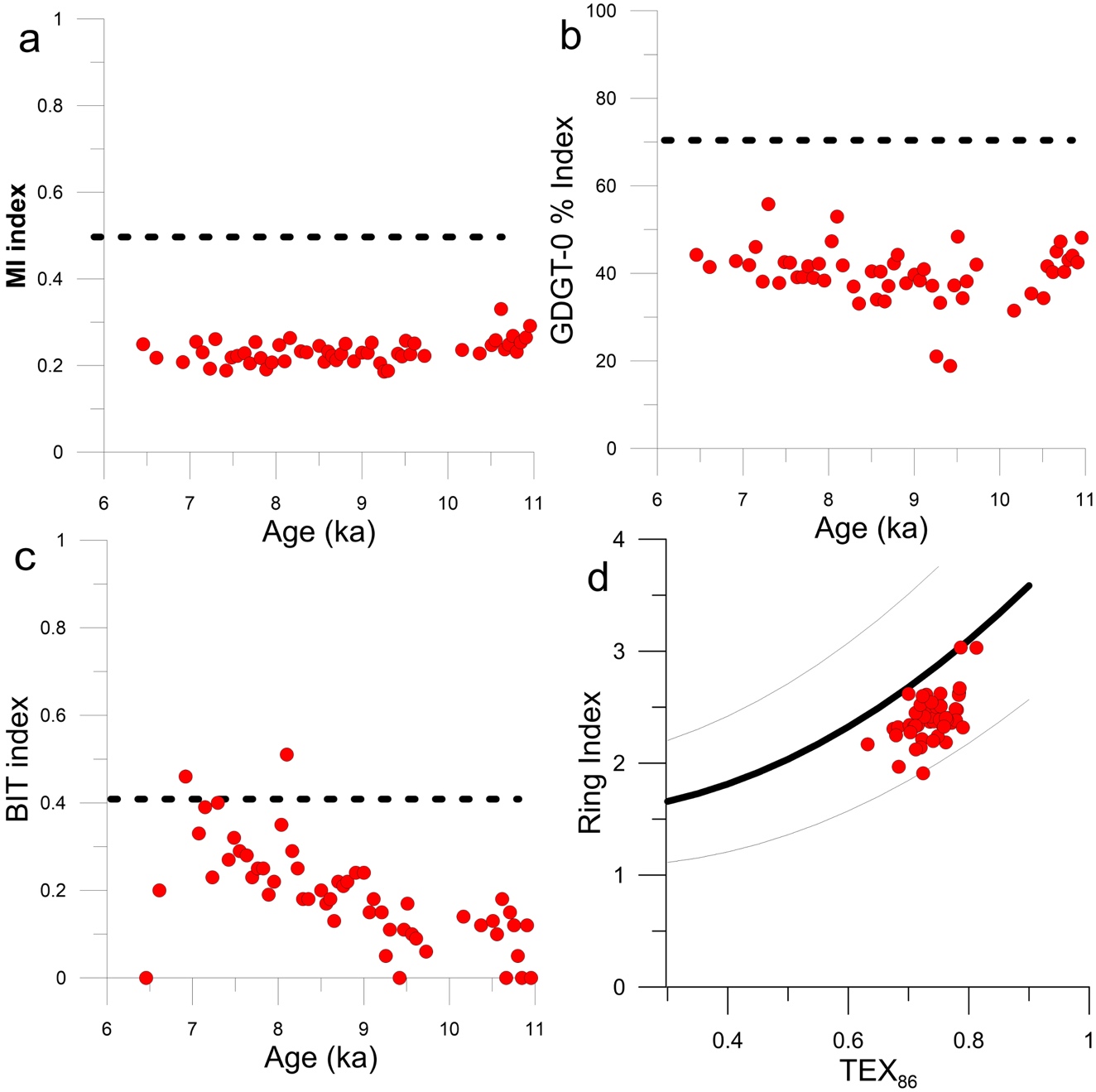


Supplemental figure 1. Figure showing the results of several TEX_86_ tests on the data. a MI index b. GDGT-0% c. BIT index d. ring index.

**2. Reanalysis of original carbonate data.**

The coral reef platform area reduction in the Late Miocene was accompanied by a carbonate platform morphology and biota transition on the analyzed southwestern margin of the Queensland Plateau ^19^. The Middle Miocene rimmed carbonate platform was dominated by a tropical photozoan skeletal assemblage, including green algae, corals, and large benthic foraminifera. Bryozoans occur as a minor component. During the Late Miocene, the platform evolved into a carbonate ramp with a skeletal assemblage dominated by bryozoans and foraminifera, including abundant large benthic foraminifera but also planktonic foraminifera ^19^. Corals and green algae are missing in these Upper Miocene deposits. Betzler ^19^ therefore concludes that the Late Miocene facies is more representative of a foramol association (sensu ^20^). This shift in the skeletal assemblage was interpreted to reflect a shift from tropical to non-tropical, warm-temperate climate conditions ^19,21–23^, partly based on the oxygen isotope record presented in Isern et al. ^22,23^.

However, Lees and Buller ^20^ already pointed out that the modern foramol skeletal assemblage is not restricted to non-tropical areas, but extends into the tropics. A fact that has since been confirmed by numerous case studies summarized e.g. in ^24^. One good example is the modern North-West Shelf of Australia, where reef growth is restricted to the coastal zone and isolated shelf-edge reefs ^25^. Most of the inner to outer carbonate ramp of the tropical North-West Shelf of Australia is characterized by heterozoan carbonates ^26^. In contrast, typical tropical carbonates including ooids and aragonite needle mud were produced and deposited during cold but arid glacial intervals in the Mid- to Late Pleistocene ^27–29^. The lack of these tropical, aragonitic components during the warmer interglacials was explained by increased runoff caused by the intensification of the Australian Monsoon ^28^, indicating the importance of environmental factors other than temperature.

There is now also a growing number of studies demonstrating that carbonate platforms dominated by foramol and other heterozoan skeletal grain associations were thriving under tropical temperature conditions during the Miocene. ^30^ demonstrate that carbonate ramps characterized by a foramol-rhodalgal grain association developed under tropical conditions during the early Late Miocene in the western Mediterranean. In this area, heterozoan grain associations generally predominated during the warmest Middle Miocene times while extensive coral reef platforms developed during the cooler climate of the latest Miocene ^30^. Tropical sea surface temperatures of > 29°C in the western Mediterranean were since confirmed by independent temperature proxies (planktonic foraminiferal Mg/Ca and UK´37) for a time window during the Middle Miocene ^31^. Pomar et al^30^ therefore conclude that factors other than temperature exerted the major control on the skeletal grain association deposited at this time. They infer that a decrease in nutrient availability was responsible for the change from a foramol-rhodalgal carbonate system to a coral reef system during the Late Miocene in the western Mediterranean ^30,32^.

Tropical coral reef systems were abundant in equatorial SE Asia during the Miocene ^33^. However, at the same time, the foramol facies was common in the deeper photic zone, in areas characterized by terrestrial runoff, or increased nutrient availability ^33^. The nutrient levels were typically in the high oligotrophy to mesotrophy range rather than eutrophic ^34^.

Another good Miocene example of heterozoan carbonates extending into the tropics was described by ^35^. They show that Early Miocene coral reef platforms were succeeded by rhodolith facies with abundant large benthic foraminifera over wide areas of the tropical Pacific in the Middle Miocene. This facies change was interpreted to reflect reef drowning due to sea-level rise in combination with a reduction of coral reef growth rates by increased nutrient levels ^35^

All of these case studies emphasized that carbonate grain associations on their own are insufficient to allow an unequivocal interpretation of paleoclimatic conditions. Westphal et al. ^24^ therefore stressed that other data, e.g. from climate proxy studies, are required to support an environmental interpretation.

Betzler et al. ^21^ and Isern et al. ^22^ cite the occurrence of *Bolboforma* in the Miocene section of Site 813 as evidence for cool water conditions. Any detailed assessment of the Bolboform record from this site is complicated by the fact that, to our knowledge, their occurrence has never been documented photographically.

*Bolboforma* belongs to a group of small (commonly < 150 µm), problematic marine calcareous microfossils that extend from the early Eocene to the late Pliocene ^36^. The classification of this group remains uncertain, and because of similarity in shape, they are often misidentified as radiolarians or benthic foraminifera ^36^. Their occurrence in the southern hemisphere is restricted to subarctic surface waters ^36^. During the Miocene, DSDP Site 590 close to the Tasman front, was the northernmost limit of their distribution in the southwest Pacific. They were not found further to the north at Site 588 at the southern margin of the Coral Sea ^36,37^. Cooke et al. ^36^ mention the anomalous cooccurrence of bolboforma with subtropical microfossil assemblages at sites north of the Subtropical Front in the Tasman Sea. They suggest that the bolboforms do not represent surface water conditions at these sites but were transported away from their source region by subsurface, intermediate depth water masses.

The presence of Large Benthic Foraminifera in Late Miocene sediments in our study area indicates temperatures greater than 18°C, which is difficult to reconcile with the presence of bolboforma as was pointed out in the original studies ^22,38^. It, therefore, is likely that the Boloboforms in the Coral Sea, similar to the Tasman Sea sites described by ^36^do not indicate cool surface water temperatures.

Betzler et al. ^21^ further argued that planktic foraminiferal assemblages presented by ^39^ would point towards non-tropical conditions at the Queensland Plateau during the Late Miocene. The interpolated maps showing the planktonic foraminiferal assemblages presented indicated fully equatorial conditions for the Queensland Plateau ^39^. However, the study of Kennett et al. ^39^ does not include any sites situated close to the Queensland Plateau. The closest one is DSDP Site 208, situated ~10° southward of ODP Site 811. The Late Miocene assemblage at this site is described as equatorial to temperate transitional ^39^. Modern-day annual average temperature at Site 208 is >3.5°C colder compared to Site 811 ^18^. To our knowledge, no planktonic foraminiferal assemblage data are available for the Upper Miocene sections of ODP Expedition 133. All planktonic foraminifers mentioned ^40^ for the Upper Miocene sections of sites from the Queensland Plateau are tropical to warm sub-tropical or cosmopolitan species according to pforams@mikrotax ^41^. We, therefore, argue that an equatorial to temperate transitional planktonic foraminiferal assemblage at DSDP Site 208 does not indicate non-tropical conditions at the Queensland Plateau during the Late Miocene.

In summary, the interpretation of non-tropical temperatures in the vicinity of the Queensland Plateau during the Late Miocene is not well supported by the observed faunal and floral assemblages. The presented new temperature proxy data rather helps to better evaluate the environmental significance of the observed change in skeletal assemblages.

Work cited

1. O’Brien, C. L. *et al.* Cretaceous sea-surface temperature evolution: Constraints from TEX 86 and planktonic foraminiferal oxygen isotopes. *Earth-Science Rev.* **172**, 224–247 (2017).

2. Hollis, C. J. *et al.* The DeepMIP contribution to PMIP4: Methodologies for selection, compilation and analysis of latest Paleocene and early Eocene climate proxy data, incorporating version 0.1 of the DeepMIP database. *Geosci. Model Dev.* **12**, 3149–3206 (2019).

3. Zhang, Y. G. *et al.* Methane Index: A tetraether archaeal lipid biomarker indicator for detecting the instability of marine gas hydrates. *Earth Planet. Sci. Lett.* **307**, 525–534 (2011).

4. Weijers, J. W. H. *et al.* Membrane lipids of mesophilic anaerobic bacteria thriving in peats have typical archaeal traits. *Environ. Microbiol.* **8**, 648–657 (2006).

5. Sinninghe Damsté, J. S., Ossebaar, J., Schouten, S. & Verschuren, D. Distribution of tetraether lipids in the 25-ka sedimentary record of Lake Challa: extracting reliable TEX86 and MBT/CBT palaeotemperatures from an equatorial African lake. *Quat. Sci. Rev.* **50**, 43–54 (2012).

6. Inglis, G. N. *et al.* Descent toward the Icehouse: Eocene sea surface cooling inferred from GDGT distributions. *Paleoceanography* **30**, 1000–1020 (2015).

7. Zhang, Y. G., Pagani, M. & Wang, Z. Ring Index: A new strategy to evaluate the integrity of TEX 86 paleothermometry. *Paleoceanography* **31**, 220–232 (2016).

8. Taylor, K. W. R., Huber, M., Hollis, C. J., Hernandez-Sanchez, M. T. & Pancost, R. D. Re-evaluating modern and Palaeogene GDGT distributions: Implications for SST reconstructions. *Glob. Planet. Change* **108**, 158–174 (2013).

9. Hernández-Sánchez, M. T., Woodward, E. M. S., Taylor, K. W. R., Henderson, G. M. & Pancost, R. D. Variations in GDGT distributions through the water column in the South East Atlantic Ocean. *Geochim. Cosmochim. Acta* **132**, 337–348 (2014).

10. Schouten, S. *et al.* An interlaboratory study of TEX 86 and BIT analysis of sediments, extracts, and standard mixtures. *Geochemistry, Geophys. Geosystems* **14**, 5263–5285 (2013).

11. Fietz, S., Martínez-Garcia, A., Huguet, C., Rueda, G. & Rosell-Melé, A. Constraints in the application of the Branched and Isoprenoid Tetraether index as a terrestrial input proxy. *J. Geophys. Res.* **116**, C10032 (2011).

12. Petrick, B. *et al.* Oceanographic and climatic evolution of the southeastern subtropical Atlantic over the last 3.5 Ma. *Earth Planet. Sci. Lett.* **492**, 12–21 (2018).

13. Meinicke, N., Reimi, M. A., Ravelo, A. C. & Meckler, A. N. Coupled Mg/Ca and Clumped Isotope Measurements Indicate Lack of Substantial Mixed Layer Cooling in the Western Pacific Warm Pool During the Last ∼5 Million Years. *Paleoceanogr. Paleoclimatology* **36**, e2020PA004115 (2021).

14. Zhang, Y. G., Pagani, M. & Liu, Z. A 12-Million-Year Temperature History of the Tropical Pacific Ocean. *Science (80-. ).* **344**, 84–87 (2014).

15. Benthien, A. & Müller, P. J. Anomalously low alkenone temperatures caused by lateral particle and sediment transport in the Malvinas Current region, western Argentine Basin. *Deep. Res. Part I-Oceanographic Res. Pap.* **47**, 2369–2393 (2000).

16. Andrews, J. C. & Clegg, S. Coral Sea circulation and transport deduced from modal information models. *Deep Sea Res. Part A. Oceanogr. Res. Pap.* **36**, 957–974 (1989).

17. Choukroun, S., Ridd, P. V., Brinkman, R. & McKinna, L. I. W. On the surface circulation in the western Coral Sea and residence times in the Great Barrier Reef. *J. Geophys. Res. Ocean.* **115**, 6013 (2010).

18. Boyer, T. P. *et al.* World Ocean Atlas 2018. *OAA National Centers for Environmental Information. Dataset.* (2018).

19. Betzler, C. Ecological controls on geometreis of carbonate platforms: Miocene / Pliocene shallow-water Microfaunas and carbonate biofacies from the Queensland Plateau (NE Australia). *Facies* **37**, 147–166 (1997).

20. Lees, A. & Buller, A. T. Modern temperate-water and warm-water shelf carbonate sediments contrasted. *Mar. Geol.* **13**, M67–M73 (1972).

21. Betzler, C., Brachert, T. C. & Kroon, D. Role of climate in partial drowning of the Queensland Plateau carbonate platform (northeastern Australia). *Mar. Geol.* **123**, 11–32 (1995).

22. Isern, A. R., McKenzie, J. A. & Feary, D. A. The role of sea-surface temperature as a control on carbonate platform development in the western Coral Sea. *Palaeogeogr. Palaeoclimatol. Palaeoecol.* **124**, 247–272 (1996).

23. Isern, A. R., McKenzie, J. A. & Müller, D. W. Paleoceanographic Changes and Reef Growth off the Northeastern Australian Margin: Stable Isotopic Data from ODP Leg 133 Sites 811 and 817 and DSDP Leg 21 Site 209. *Proc. Ocean Drill. Program, 133 Sci. Results* (1993) doi:10.2973/ODP.PROC.SR.133.230.1993.

24. Westphal, H., Halfar, J. & Freiwald, A. Heterozoan carbonates in subtropical to tropical settings in the present and past. *Int. J. Earth Sci.* **99**, 153–169 (2010).

25. Collins, L. B. & Testa, V. Quaternary development of resilient reefs on the subsiding kimberley continental margin, Northwest Australia. *Brazilian J. Oceanogr.* **58**, 67–77 (2010).

26. James, N. P., Bone, Y., Kyser, T. K., Dix, G. R. & Collins, L. B. The importance of changing oceanography in controlling late Quaternary carbonate sedimentation on a high-energy, tropical, oceanic ramp: north-western Australia. *Sedimentology* **51**, 1179–1205 (2004).

27. Gallagher, S. J. *et al.* The enigma of rare Quaternary oolites in the Indian and Pacific Oceans: A result of global oceanographic physicochemical conditions or a sampling bias? *Quat. Sci. Rev.* **200**, 114–122 (2018).

28. Hallenberger, M. *et al.* Increased fluvial runoff terminated inorganic aragonite precipitation on the Northwest Shelf of Australia during the early Holocene. *Sci. Rep.* **9**, 18356 (2019).

29. Hallenberger, M. *et al.* Climate and sea‐level controlling internal architecture of a Quaternary carbonate ramp (Northwest Shelf of Australia). *Sedimentology* (2021) doi:10.1111/SED.12948.

30. Pomar, L., Brandano, M. & Westphal, H. Environmental factors influencing skeletal grain sediment associations: a critical review of Miocene examples from the western Mediterranean. *Sedimentology* **51**, 627–651 (2004).

31. Badger, M. P. S. *et al.* CO2 drawdown following the middle Miocene expansion of the Antarctic Ice Sheet. *Paleoceanography* **28**, 42–53 (2013).

32. Pomar, L. Ecological control of sedimentary accommodation: evolution from a carbonate ramp to rimmed shelf, Upper Miocene, Balearic Islands. *Palaeogeogr. Palaeoclimatol. Palaeoecol.* **175**, 249–272 (2001).

33. Wilson, M. E. J. Global and regional influences on equatorial shallow-marine carbonates during the Cenozoic. *Palaeogeogr. Palaeoclimatol. Palaeoecol.* **265**, 262–274 (2008).

34. Wilson, M. E. J. & Vecsei, A. The apparent paradox of abundant foramol facies in low latitudes: Their environmental significance and effect on platform development. *Earth-Science Rev.* **69**, 133–168 (2005).

35. Bourrouilh-Le Jan, F. G. & Hottinger, L. C. Occurrence of rhodolites in the tropical Pacific — a consequence of Mid-Miocene paleo-oceanographic change. *Sediment. Geol.* **60**, 355–367 (1988).

36. Cooke, P. J., Nelson, C. S., Crundwell, M. P. & Spiegler, D. Bolboforma as monitors of Cenozoic palaeoceanographic changes in the Southern Ocean. *Palaeogeogr. Palaeoclimatol. Palaeoecol.* **188**, 73–100 (2002).

37. Grützmacher, U. Die Veränderungen der Paläogeographischen Verbreitung von Bolboforma-ein Beitrag zur Rekonstruktion und Definition von Wassermassen im Tertiär. (1993).

38. Betzler, C. & Chaproniere, G. C. H. Paleogene and Neogene Larger Foraminifers from the Queensland Plateau: Biostratigraphy and Environmental Significance. *Proc. Ocean Drill. Program, 133 Sci. Results* (1993) doi:10.2973/ODP.PROC.SR.133.210.1993.

39. Kennett, J. P., Keller, G. & Srinivasan, M. S. Miocene planktonic foraminiferal biogeography and paleoceanographic development of the Indo-Pacific region. *The Miocene Ocean: Paleoceanography and Biogeography. Geological Society of America Memoir* vol. 163 197–236 (1985).

40. Davies, P. J., Mckenzie, J. A., Palmer-Julson, A. & Shipboard Scientific Party. *Sites 811/825*. vol. 133 (Ocean Drilling Program, 1991).

41. Young, J. R., Wade, B. S. & Huber B.T. pforams@mikrotax website. *URL: http://www.mikrotax.org/pforams* https://www.mikrotax.org/pforams/pf-pages/pf-citation.html (2017).
